# Glioblastoma Multiforme Involving Conus Medullaris in a Child

**DOI:** 10.7759/cureus.2863

**Published:** 2018-06-22

**Authors:** Muhammad Atif Mansha, Agha Muhammad Hammad Khan, Ahmed Nadeem N Abbasi, Muhammad Usman U Tariq, Naureen Mushtaq, Maria Tariq, Asmara Waheed

**Affiliations:** 1 Oncology, Aga Khan University, Karachi, PAK; 2 Pathology & Laboratory Medicine, Aga Khan University, Karachi, PAK; 3 Pediatrics, Aga Khan University, Karachi, PAK

**Keywords:** pediatric neuro-oncology, spinal tumor

## Abstract

Primary spinal cord glioblastoma multiforme involving the conus medullaris is an uncommon entity with poor outcomes. An aggressive multimodality treatment approach has been used, but prognosis remains same. There are no guidelines for the treatment of patients with spinal glioblastoma multiforme (GBM).

We highlight the case of a child diagnosed with conal GBM. He was treated with definitive surgery followed by adjuvant concurrent chemoradiation. After completion of treatment, he showed a temporary symptomatic improvement, but later on his condition deteriorated. We elaborate the stepwise treatment approach employed in this patient.

## Introduction

Spinal cord tumors in the pediatric population are rare, featuring less than 1% of all central nervous system tumors [1]. Of these, up to 35% of all spinal tumors in children are intramedullary [2]. Astrocytomas are, by far, the most common spinal cord tumors in children whereas in adults ependymomas represent the majority of intramedullary tumors [3].

Spinal cord glioblastoma multiforme (GBM) accounts for approximately 7.5% of all intramedullary gliomas and 1.5% of all spinal cord tumors [1, 4]. It has a predilection for development in the cervical or cervicothoracic region [5]. GBM involving conus medullaris is an uncommon entity that carries a poor prognosis [6]. Children usually complain of pain as the presenting symptom, which has been reported in 42% of cases [3].

The aim of this paper is to present a case of a 15-year-old boy with intramedullary conal GBM and to discuss the clinical findings and therapeutic interventions used to treat this tumor. We present a review of the relevant literature.

## Case presentation

A 15-year-old boy, grade 9 student, presented in neurosurgery clinic with complaints of backache and left leg numbness. According to the patient’s father, his symptoms started three months prior when he developed pain in the lower back. Symptoms were gradual in onset, continuous and progressively increasing in intensity from moderate to severe. The pain was worse at night and was relieved by taking paracetamol (acetaminophen). It was also associated with weakness in the lower limbs, with a left-sided predominance. A week prior to presentation, the patient developed urinary retention and constipation. His birth and family histories were insignificant. Vaccination and developmental milestones were up to date. General physical exam was unremarkable. Systemic examination revealed decreased power in the lower limbs, bilaterally positive straight leg response and absent plantar reflexes.

**Figure 1:**
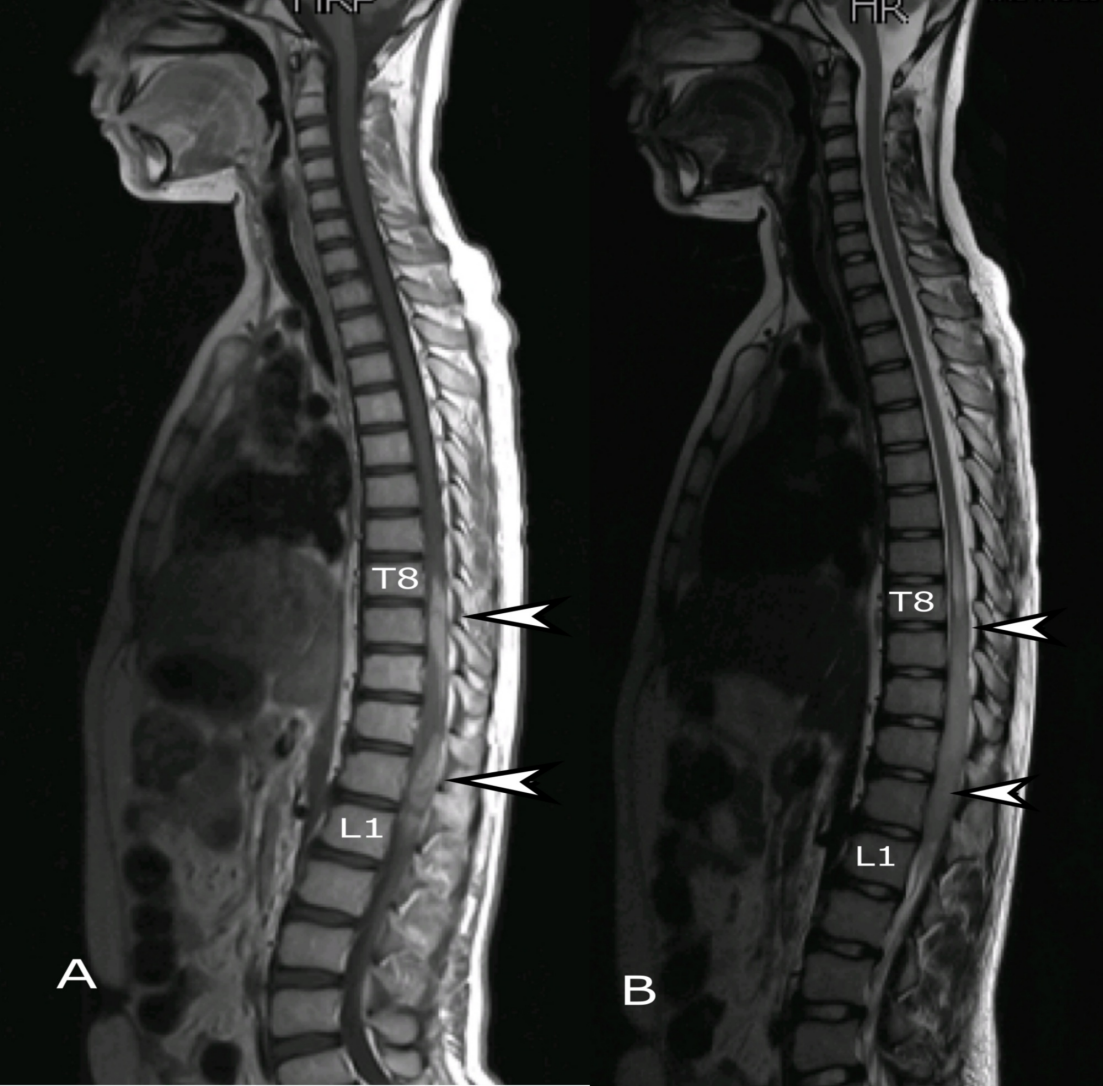
MRI whole spine, sagittal view. (A) T2 weighted and (B) T1 weighted post-contrast images showing intramedullary lesion (arrows) extending from T8 to L1. MRI: Magnetic resonance imaging.

**Figure 2:**
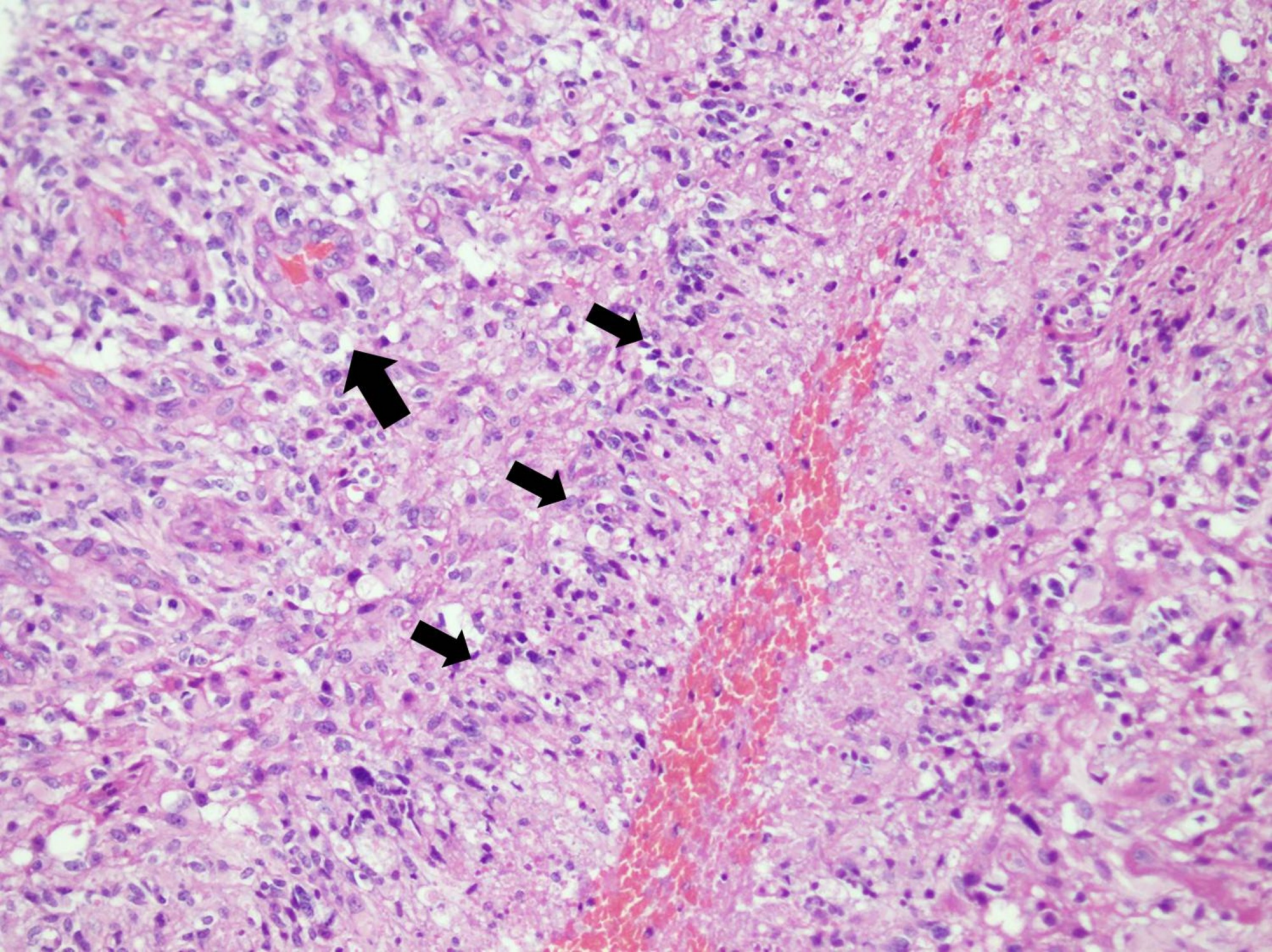
Low power view of cellular glial neoplasm arranged in sheets of pleomorphic neoplastic cells. Glomeruloid vascular proliferation (long arrow head) and palisading necrosis is also seen (short arrow heads). (H&E stain; 100x magnification)

**Figure 3:**
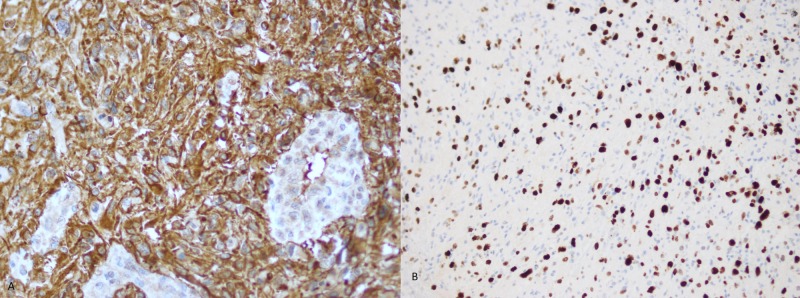
(A) Tumor cells show diffuse positive expression for GFAP immunohistochemical stain and (B) High Ki-67 (Mib-1) index. GFAP: Glial fibrillary acidic protein.

Considering the presenting complaints and examination findings, the patient was admitted for further workup. Magnetic resonance imaging (MRI) of the whole spine was performed which revealed an intramedullary lesion extending from T8 to L1 vertebrae involving the conus. The maximum dimension of the lesion was 138 mm (Figure 1). A decompression laminectomy for excision of space occupying lesion was performed. The resected specimen was sent for histopathological review, where the diagnosis of glioblastoma multiforme was established (Figures 2, 3).

**Figure 4:**
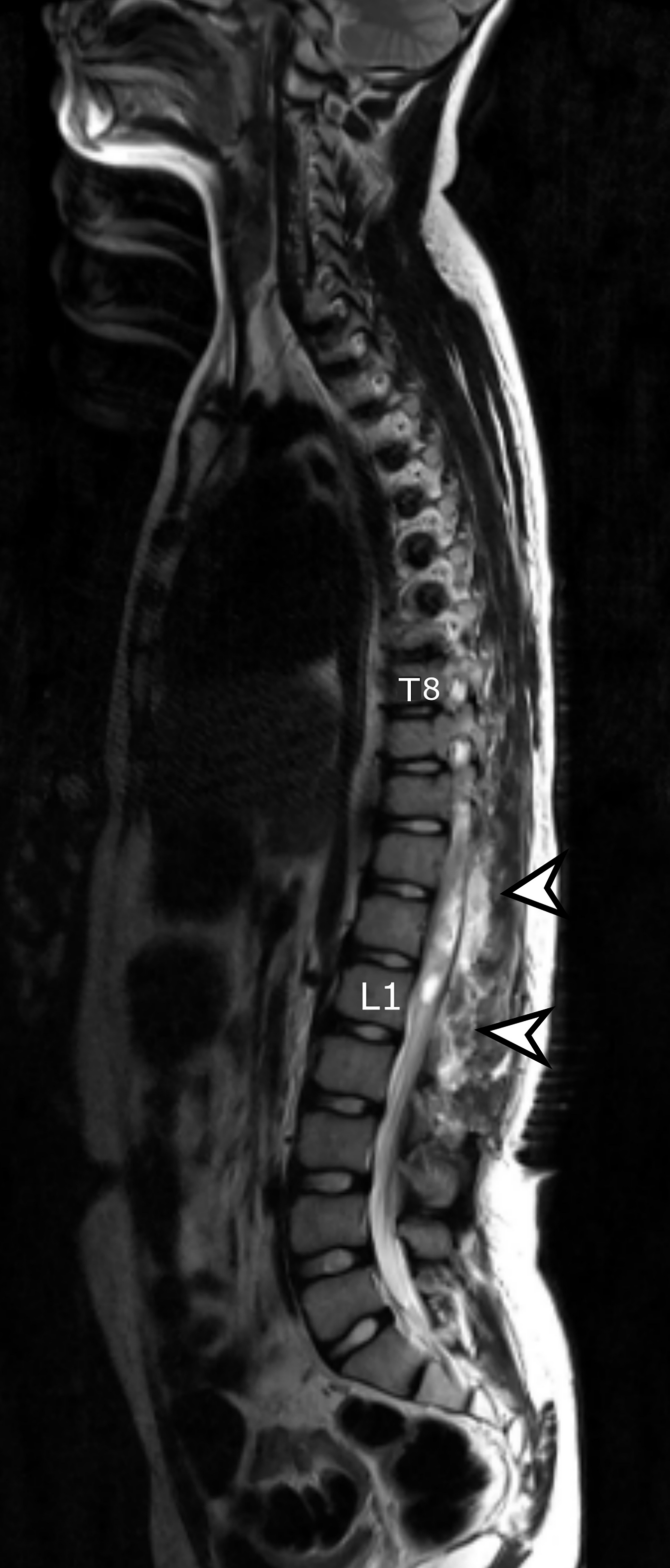
Immediate post-operative MRI spine, sagittal view. T2 weighted image showing hyperintense signals identified extending proximally in the thoracic spinal cord representing cord edema due to post-surgical changes (arrows). MRI: Magnetic resonance imaging.

Immediate post-operative MRI of thoracic and lumbar spine demonstrated post-surgical changes along with hemorrhage at the site of surgery with cord edema (Figure 4). MRI brain showed no metastatic disease. Post-operatively, the patient had noticeably reduced sensation and power in the lower limbs, making him bedbound. Physical rehabilitation was then instituted which improved his condition slightly to the extent that he could be mobilized using a wheelchair.

**Figure 5:**
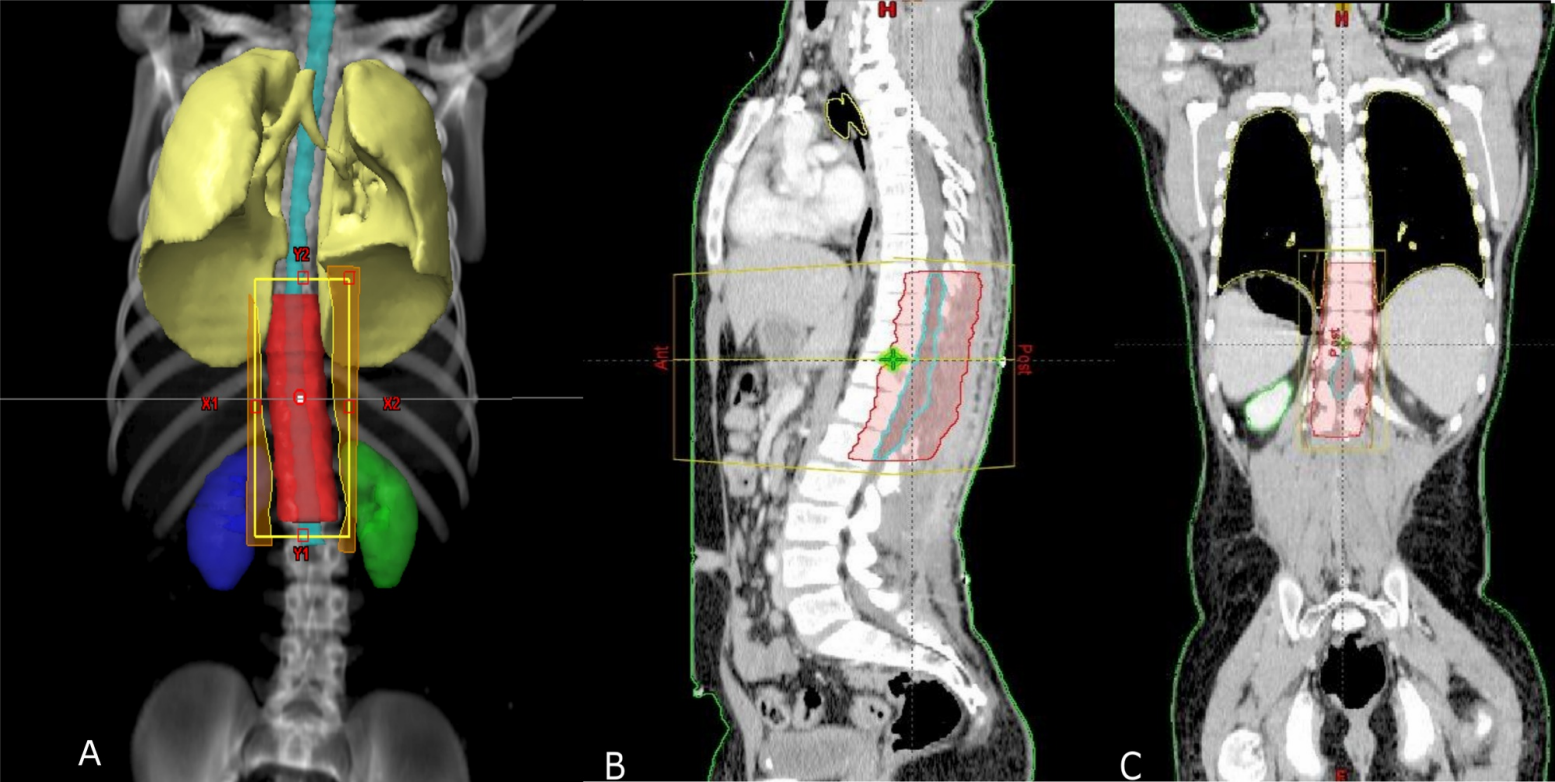
Radiation treatment plan. (A) Three-dimensional conformal radiation treatment plan showing digitally reconstructed radiograph. (B) Sagittal and (C) coronal views showing primary target volume in red.

The case was further discussed in site-specific multidisciplinary team meeting, where the consensus was to offer adjuvant concurrent chemo-radiation (CCRT). A total radiation dose of 4500 cGy in 25 fractions at 180 cGy per fraction per day was given to the tumor bed along with concomitant temozolomide at a dose of 75 mg/m^2^ (Figure 5). During the course of treatment, the patient was examined weekly for any treatment-related side effects. After completion of CCRT, maintenance chemotherapy with temozolomide at a dose of 150 mg/m^2^ was continued.

**Figure 6:**
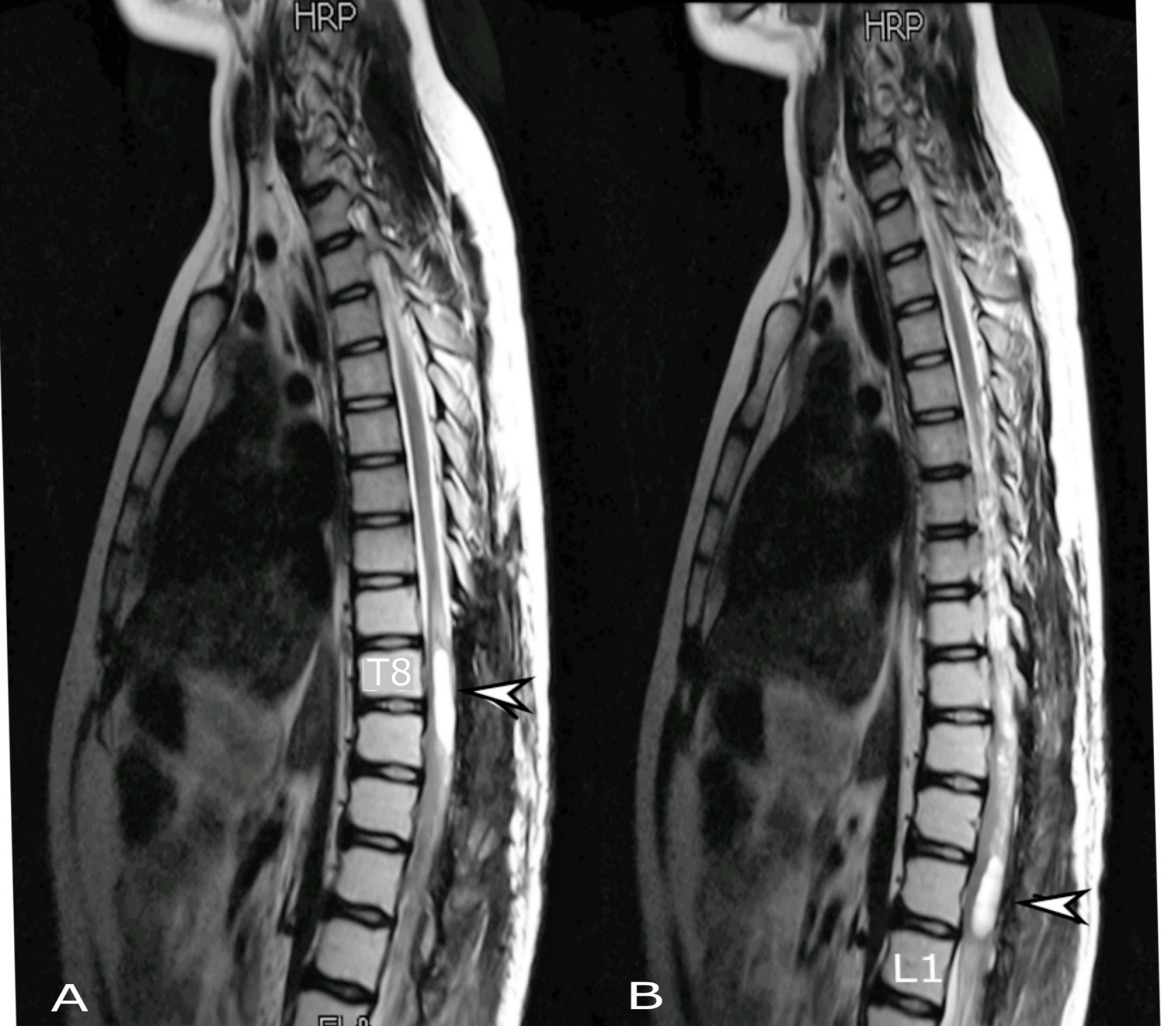
Post-radiation MRI thoracic spine, sagittal section. T2 weighted image showing development of cystic degeneration at (A) T8-T9 and (B) T12-L1 (arrows). MRI: Magnetic resonance imaging.

Six weeks post CCRT, the patient was reviewed in the clinic. There was clinically significant improvement in power of the lower limbs. MRI of the whole spine revealed interval development of cystic degeneration with peripheral enhancement in the irradiated area along with cord expansion and edema (Figure 6). Maintenance chemotherapy with temozolomide was continued and he was advised to follow in the clinic after three months along with a repeat MRI of the craniospinal axis.

**Figure 7:**
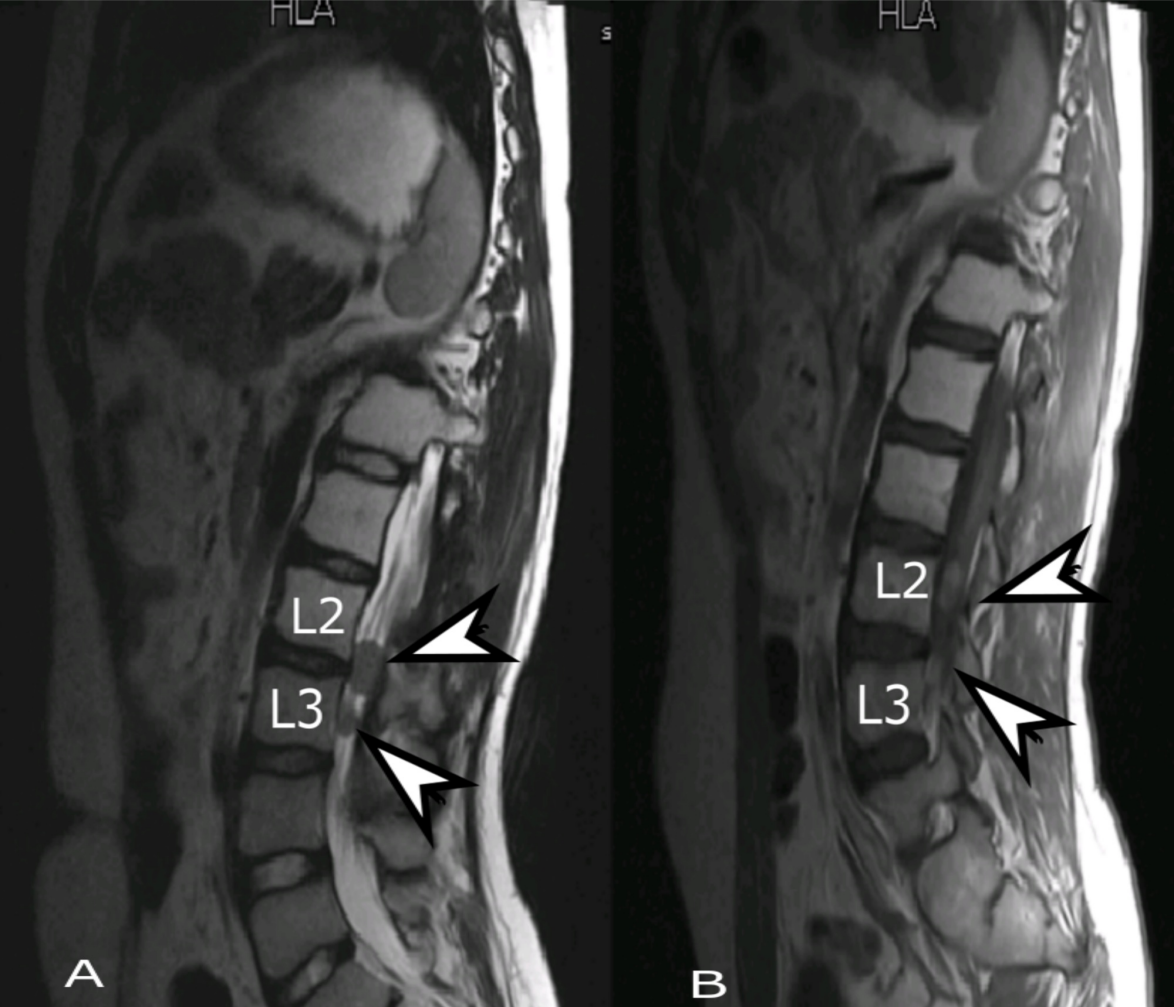
Follow-up MRI lumbar spine, sagittal view. (A) T2 weighted and (B) T1 weighted post-contrast images showing interval development of two new rounded lesions (arrows). MRI: Magnetic resonance imaging.

On the subsequent follow-up, the patient complained of headache and pain in lower back. On examination, there was a substantial decline of power in the lower limbs. MRI showed interval development of two new rounded lesions at L2-L3 vertebral levels which were suggestive of disease progression (Figure 7). The patient was then referred to palliative care team for further management. As pain was gradually controlled, he was discharged home on pain medications. A month later he passed away due to disease progression.

## Discussion

Primary intramedullary tumors in the spinal cord are rare entities [7]. The most common intramedullary neoplasm in children is astrocytoma followed by ependymoma [8]. Spinal gliomas occur at a relatively younger age with a slight predilection for the second and third decades of life [6]. Despite using multiple modalities of treatment, these tumors are generally associated with a dismal outcome, with a median survival of 15 months [4, 9-11].

Review of the literature demonstrates that spinal cord GBM occurs mainly in the cervical or thoracic segments [5, 10, 12, 13]. Clinical presentation depends upon the site and extent of the spinal cord involvement with the most commonly reported symptom in children being pain [2, 14]. At the time of presentation, our patient had complaints of worsening lower backache, numbness in left lower limb, progressive paraparesis and a week-long history of urinary retention and constipation, which raised a suspicion of conus involvement. Mathew and Todd published a review of 62 patients with tumor involving conus and cauda equina. Tumors invading conus medullaris were more likely to cause sphincter dysfunction and interestingly, in half of the patients, there was no back pain [15].

Radiology and histopathology are essential to establish the diagnosis of GBM. MRI is considered the gold standard imaging technique to diagnose intramedullary tumors [1, 14]. Caroli et al. recommend evaluation of the entire craniospinal axis to rule out any synchronous disease [8]. In the presented case, MRI precisely defined the tumor extending from T8 to L1. The histopathological features of spinal-GBM are similar to intracranial GBM including cellular pleomorphism, high mitotic activity, necrosis and vascular proliferation. There is an established role of cerebrospinal fluid cytology with positive cytology conferring a poor outcome indicating its prognostic value [4].

An aggressive therapeutic approach is recommended considering the likelihood of early spread and poor prognosis of spinal GBM. Available modalities include surgery, radiation therapy and chemotherapy. A systematic review conducted by Konar et al. has shown that surgery remains the definitive modality in spinal glioblastoma [9]. Due to its infiltrative nature, there is a surgical limitation of a clear cleavage plane between the diseased and the normal medullary parenchyma, therefore a gross total resection is not achievable. Hence, a maximum safe resection is a preferred approach in order to avoid post-surgical complications. Moreover, the overall survival is not altered even if a gross total resection is achieved [8, 16, 17].

Subject to the rarity of this tumor, the optimum adjuvant therapy is not well defined, but the addition of radiotherapy provides better clinical outcomes and may prolong the survival in some cases [18]. Radiation doses of up to 50.4 Gy have been reported in the literature [19]. Higher radiation doses will exceed the tolerance limits of the normal spinal cord.

Similarly, the role of chemotherapy is not well established due to the paucity of cases, yet, most of the children with high-grade disease receive temozolomide with little improvement in outcomes [20]. The ideal time of starting radiation and chemotherapy is questionable but usually a span of four to six weeks after surgery is reasonable [17, 18]. Chemotherapy drugs other than temozolomide have also been tried but failed to show a survival advantage [17].

With our case report, we have documented all events with relation to time, which will help to further validate the prognosis and treatment outcome of this rare entity.

## Conclusions

Spinal cord GBM involving the conus medullaris carries a likely lethal prognosis with a short survival. There is a need for a multidisciplinary approach for the management of such patients. Even with the most aggressive treatment approaches involving surgery, radiation and chemotherapy, the outcome does not improve. Aggressive natural history, rarity and lack of management guidelines mandate a thorough discussion in site-specific multidisciplinary team meeting before embarking on to any modality of treatment.
